# De Synchondrotomia Pubis Commentarius

**Published:** 1783

**Authors:** 


V. Jani Peterson Michell, M. D. *De Synchondrotomia Pubis Commentarius.* 8vo. Amstelodami, 1783. P. 260, with two copper-plates.

THIS essay exhibits a very accurate review of every thing that has been written on the Continent for or against the section of the Symphysis Pubis.

The work is divided into three sections, each of which is subdivided into chapters. In the first section the author gives the history of the new operation; and in the second he candidly discusses its proposed advantages, and from a review of the different cases in which it has been performed, endeavours to prove its inutility and danger. Dr. Osborn, in his late publication on laborious parturition *, has given a very full account of all these cases, excepting two that occurred in Holland, and of which he had not been able to learn the particulars at the time his essay was printed. In both these instances the operation was performed by Mr. Van Munster, surgeon, at Bommel. The first of the two patients was a ricketty women, 27 years old,

* See p. 35.

who who had formerly been delivered of a dead child at the full time. In this cafe the diameter of the Pelvis, at its brim, from the Pubis to the Os Sacrum, was eftimated at two inches and three quarters. The operation was performed on the 26th of March 1773. The Symphyfis Pubis was divided with a knife, without any injury being done to the Clitoris or Vagina. The bones immediately feparated, and with the affiftance firft of a blade of the forceps and afterwards of the finger, a fpace of two inches was obtained between them. The head of the fœtus, which was in a putrid ftate, foon got lower down, and the labour was eafily terminated with the forceps.—Two days after the operation the bones of the Pubis were ftill half an inch diftant from each other, but the feparation became gradually lefs and lefs, and, on the 6th of April was hardly perceptible.—The patient voided her urine and ftools without any difficulty, and at the end of fix weeks was able to walk. But there ftill remained a fiftulous fore at the Pubis, which was feveral months before it healed, and even now, we are told, the bones are not perfectly united, fo that fhe cannot walk with firmnefs.

Mr.

Mr. Van Munſter's ſecond patient was likewiſe ricketty, and nearly of the ſame age as the firſt. She underwent the operation in her ſecond labour, on the 8th of April, having been delivered of a dead child in her firſt pregnancy with the aſſiſtance of the forceps. The diameter of the Pelvis, in this caſe, we are told, was hardly three inches. The ſection was performed with the ſame ſucceſs as in the former patient. A ſpace of an inch and a half was procured, and the woman was delivered of a live child with Levret's forceps.——She voided her urine freely after the operation, but on the third day was attacked with fever, which continued till the tenth. During the firſt fourteen days ſhe was obliged to lie conſtantly on her back, but after that time ſhe was able to lie on either ſide. In a month ſhe was ſufficiently recovered to get out of bed, and at the end of ſix weeks was able to walk. A fiſtulous opening remained, as in the former caſe, for the ſpace of four months, and the bones of the Pubis threw off a ſmall exfoliation. There is ſtill a ſpace of half an inch at the upper part of the Symphyſis, but at the lower part the bones are firmly united, ſo that the patient feels no inconvenience from the operation. But ſhe

ſo much dreads a repetition of it, that ſince her recovery ſhe has conſtantly refuſed to admit the embraces of her huſband.

The event of the operation in theſe caſes has perhaps been more ſucceſsful than in any in which it has been performed, but the author of the work before us ſeems very properly to obſerve, that the ſection of the Symphyſis was in neither of theſe two patients neceſſary.

In the third ſection of his work, the author treats of the Cæſarean operation, for which he is a zealous advocate, in preference to the diviſion of the Symphyſis.

As a proof of the injury that may with impunity be offered to the Uterus, he mentions a ſingular caſe in which Mr. Van Munſter, the ſurgeon already mentioned, being induced to think that the rigidity of the Os Uteri was occaſioned by a cicatrix formed after a laceration of it in a former labour, boldly ventured to dilate it with a knife, and yet the patient afterwards recovered perfect health. But the eagerneſs, (perhaps, without uſing too ſtrong an expreſſion, we may ſay intemperance) with which our author recommends the dreadful expedient of Hyſterotomy, in caſes of narrow Pelvis, ſeems to be in a great meaſure founded on a recent inſtance

 of of its ſucceſs at Leyden, and which has already been curſorily mentioned in a former part* of this volume. The ſubject of this extraordinary caſe was in her 26th year. We are told that the Cæſarean ſection was deemed neceſſary on account of the extreme narrowneſs of the Pelvis, but its particular dimenſions are not mentioned. The operation was performed on the 26th of January 1782, by Dr. Brand. An inciſion of ſeven inches in length was made into the Uterus on the right ſide of the Abdomen. The hæmorrhage was conſiderable, and a male fœtus alive and of a large ſize was extracted. The Placenta was then carefully removed, and the wound having ſoon contracted to the length of an inch and a half, the hæmorrhage ceaſed, and at the end of four hours the Lochia began to paſs through the Os Uteri. On the ſixth day, when the wound was beginning to ſuppurate, the patient having been guilty of ſome irregularity in her diet, was ſeized with a vomiting, and, in ſtraining, burſt open the ſuture by which the lips of the wound were united. About the ſame time likewiſe an abſceſs began to form, which our author aſcribes to a meſtaſtaſis of the milk.

The matter was evacuated by puncturing the Vagina.—Notwithstanding these unfavourable circumstances, the patient, before two months had elapsed, was so well recovered as to be able to go to church, which she did on the 24th of March. She is said to be now perfectly free from hernia, and has been again pregnant since the operation, but miscarried.

